# Genomics, Computational Biology and Drug Discovery for Mycobacterial Infections: Fighting the Emergence of Resistance

**DOI:** 10.3389/fgene.2020.00965

**Published:** 2020-09-04

**Authors:** Asma Munir, Sundeep Chaitanya Vedithi, Amanda K. Chaplin, Tom L. Blundell

**Affiliations:** Department of Biochemistry, University of Cambridge, Cambridge, United Kingdom

**Keywords:** emergence of resistance, tuberculosis, leprosy, prediction of mutations, antimycobacterials

## Abstract

Tuberculosis (TB) and leprosy are mycobacterial infections caused by *Mycobacterium tuberculosis* and *Mycobacterium leprae* respectively. These diseases continue to be endemic in developing countries where the cost of new medicines presents major challenges. The situation is further exacerbated by the emergence of resistance to many front-line antibiotics. A priority now is to design new antimycobacterials that are not only effective in combatting the diseases but are also less likely to give rise to resistance. In both these respects understanding the structure of drug targets in *M. tuberculosis* and *M. leprae* is crucial. In this review we describe structure-guided approaches to understanding the impacts of mutations that give rise to antimycobacterial resistance and the use of this information in the design of new medicines.

## Mycobacterial Infections in Tuberculosis and Leprosy

Mycobacterial infections in tuberculosis [(WHO Global Tuberculosis Report, 2017] and leprosy (Chaptini and Marshman, 2015; WHO, 2019) are both endemic in developing countries. According to the World Health Organization the risk of developing TB is estimated to be between 16 and 27 times greater in people living with HIV than among those without HIV infection. TB is a major challenge in developing countries such as India and South Africa. Similar challenges are evident in the fight against leprosy, particularly in India, Brazil and Indonesia. However, the stigma associated with leprosy and the confinement of those affected to leper colonies have led to less public discussion of strategies to combat the disease.

Current anti-tuberculosis therapies use a combination of front-line drugs, such as isoniazid (INH), rifampin, ethambutol (EMB), pyrazinamide (PZA) and streptomycin (SM), most of which were discovered five or six decades ago. These have led to improvements in health with as many as 90% of patients cured. However, the fact that these drugs must be taken for 6 months often leads to non-compliance, resulting in further spread of the disease and the development of drug resistance. In the case of tuberculosis multidrug-resistant (MDR)-TB (resistant to INH and rifampin) and extensive-drug-resistant (XDR)-TB strains require the use of second-line drugs that are much more toxic and expensive (Wallis et al., 2016; Munir et al., 2019). This has been further complicated by the HIV over the past four decades.

Fighting leprosy involves even greater challenges. *M. leprae*, the causative bacillus for leprosy, continues to be endemic in populations in some tropical and sub-tropical countries, including India, Brazil, Indonesia and parts of Africa. The fact that *M. leprae* is non-cultivable makes it difficult to work with within a laboratory setting. This has led to poor understanding of the genomic diversity and the structural organization of the multicomponent protein systems that mediate host cell invasion and pathogenesis. Introduction of multidrug therapy (Dapsone, rifampin and Clofazimine) has reduced global numbers from 5 million cases in 1995 to approximately 200,000 cases in 2005 (Chaptini and Marshman, 2015). Since then, the number of incidences has remained stable, and less effort has been focused on patients infected with *M. leprae* in recent years (Duthie et al., 2018), partly due to a lack of awareness of the extent of the disease. Leprosy manifests as skin lesions and demyelinating neuropathy leading to numbness, tissue deformity and blindness (WHO, 2019). In the absence of a vaccine, the drugs currently used have been repurposed from those used for TB.

The situation with both tuberculosis and leprosy is exacerbated by the emergence of antibiotic resistance to all components of the multi-drug therapy (Williams and Gillis, 2012). However, traditional approaches to discovery of new antimycobacterials, such as phenotypic screens, which do not attempt to identify the targets of potential new drugs, have exhibited very low hit rates. This is likely due to the limited chemical diversity of the compound libraries, but also may be a result of a focus on drug-like molecules and therefore the omission of smaller and larger molecules that may more efficiently penetrate the mycobacterial cell wall (Payne et al., 2007; Koul et al., 2011). On the other hand high-throughput, target-based screening campaigns have often appeared successful *in vitro*, but this is not always reflected *in vivo.* Clinical trials of potential drugs have also highlighted increased challenges in finding suitable candidates. This is often due to the complexity of replication states in *M. tuberculosis* and the variety of lesions present within patients, neither of which are adequately addressed within the *in vitro* screening (Zumla et al., 2013; Prideaux et al., 2015). As a consequence, there has been a focus on alternative approaches, including the use of natural products and drug repurposing.

In an earlier review article (Waman et al., 2019), we discussed various computational approaches and experimental strategies for drug target identification and structure-guided drug discovery. In this review we discuss the impact of the era of precision medicine, where the genome sequences of pathogens can give clues about the choice of existing drugs, and repurposing of others. Our focus is directed toward combatting antimicrobial drug resistance with emphasis on tuberculosis and leprosy. We describe structure-guided approaches to understanding the impacts of mutations that give rise to antimycobacterial resistance and the use of this information in the design of new medicines.

## Genome Sequences and Proteomic Structural Databases

In recent years, there have been many focused efforts to define the amino-acid sequences of the *M. tuberculosis* pan-genome and then to define the three-dimensional structures and functional interactions of these gene products. This work has led to essential genes of the bacteria being revealed and to a better understanding of the genetic diversity in different strains that might lead to a selective advantage (Coll et al., 2018). This will help with our understanding of the mode of antibiotic resistance within these strains and aid structure-guided drug discovery. However, only ∼10% of the ∼4128 proteins have structures determined experimentally.

Several databases have been developed to integrate the genomic and/or structural information linked to drug resistance in Mycobacteria (Table 1). These invaluable resources can contribute to better understanding of molecular mechanisms involved in drug resistance and improvement in the selection of potential drug targets.

**TABLE 1:** List of databases/resources for structural and/or mutational analyses in Mycobacteria.

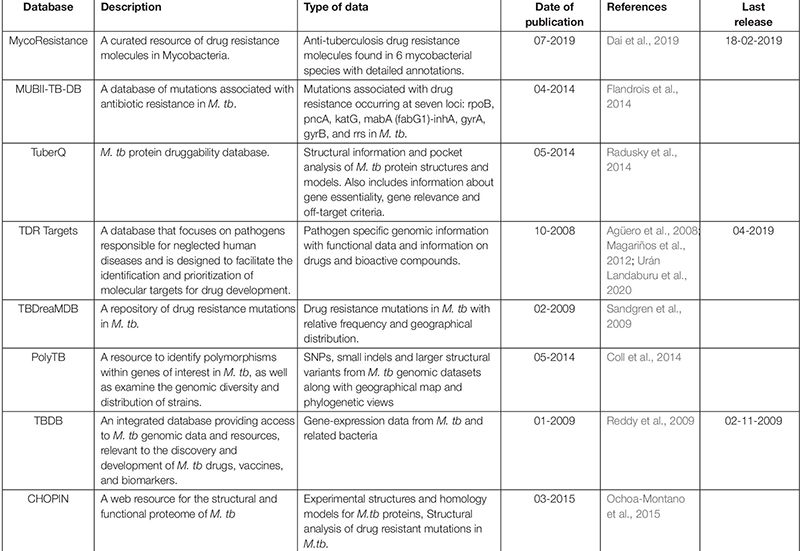

Our own laboratory has developed a database, CHOPIN (Ochoa-Montano et al., 2015), which records experimental structures of the proteins. It then adds homology models developed by techniques, for example FUGUE for sequence-structure alignment (Shi et al., 2001) and MODELER for comparative modeling by satisfaction of spatial restraints (Sali and Blundell, 1993), to accumulate information about structures of ∼3000 gene products. This corresponds to ∼73% of the proteome. These models have been elaborated using a complex pipeline to reflect different functional states of the proteins, characteristics of different oligomeric states and ligand binding. Additionally, CHOPIN includes structural analyses of mutations potentially associated with drug resistance. Results are made available at the web interface, which also serves as an automatically updated repository of all published TB experimental structures.

*Mycobacterium leprae* has a reductively evolved genome of 1,614 protein coding genes (Singh and Cole, 2011), of which 595 code for hypothetical proteins and 1,010 for proteins with functional assignments. Most of these are annotated by homology with phylogenetically related species in the family of *Mycobacteriaceae*. The first analysis was of the 3.26Mb genome of the TN strain of *M. leprae* which was sequenced from an armadillo-derived Indian isolate in 2001 (Cole et al., 2001). The genome contains 49.5% protein coding regions and 27% of inactive reading frames that have functional orthologs in *M. tuberculosis*. The rest of the genome contains either regulatory elements or repetitive non-coding regions. The average G + C content of the genome is 57.8% (Cole et al., 2001). In a more recent study ∼1,310 pseudogenes were identified in the genome of *M. leprae* (Chavarro-Portillo et al., 2019).

There is a dearth of information related to structural aspects of proteins from *M. leprae* and their oligomeric and hetero-oligomeric organization, which has limited the understanding of physiological processes of the bacillus. The structures of only 12 proteins have been solved and deposited in the protein data bank (PDB). However, the high sequence similarity in protein coding genes between *M. leprae* and *M. tuberculosis* allows computational methods to be used for comparative modeling of the proteins of *M. leprae.* Mainly monomeric models using single template modeling have been defined and deposited in the Swiss Model repository (Bienert et al., 2017), in Modbase (Pieper et al., 2014), and in a collection with other infectious disease agents (Sosa et al., 2018). There is a need for multi-template modeling and building homo- and hetero-oligomeric complexes to better understand the interfaces, druggability and impacts of mutations.

**FIGURE 1:**
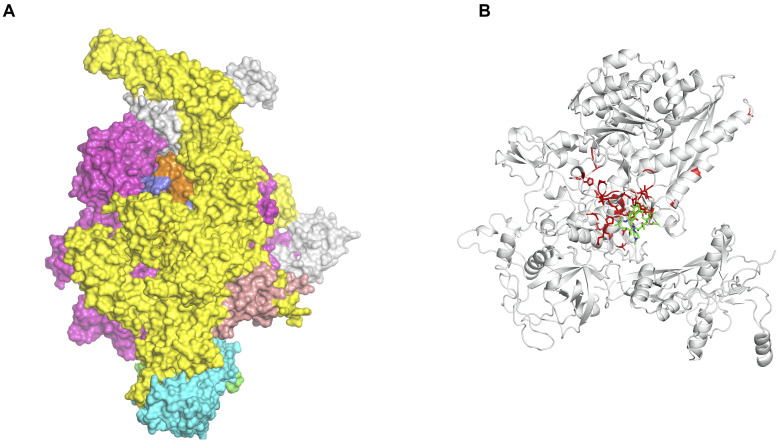
**(A)** The hetero-hexameric model of the holoenzyme complex of RNA polymerase in *Mycobacterium leprae*. The colors represent each chain in the model (β, β′, α′, α′′, ω, σ subunits) (Vedithi et al., 2018). **(B)** The ß-subunit of RNA polymerase (RpoB) with mutations (highlighted in red). Mutations at these residue positions are known to confer rifampin resistance in leprosy. Rifampin is colored in green (Vedithi et al., 2020).

We are now exploiting Vivace, a multi-template modeling pipeline developed in our lab for modeling the proteomes of *M. tuberculosis* (CHOPIN, see above) and *M. abscessus* [Mabellini Database (Skwark et al., 2019)], to model the proteome of *M. leprae*. We emphasize the need for understanding the protein interfaces that are critical to function. An example of this is that of the RNA-polymerase holoenzyme complex from *M. leprae*. We first modeled the structure of this hetero-hexamer complex and later deciphered the binding patterns of rifampin (Vedithi et al., 2018; Figures 1A,B). Rifampin is a known drug to treat tuberculosis and leprosy. Owing to high rifampin resistance in tuberculosis and emerging resistance in leprosy, we used an approach known as “Computational Saturation Mutagenesis”, to identify sites on the protein that are less impacted by mutations. In this study, we were able to understand the association between predicted impacts of mutations on the structure and phenotypic rifampin-resistance outcomes in leprosy.

## Drug Discovery Using Structure-Guided Fragment-Based Approaches

Structure-guided fragment-based drug discovery (FBDD) is a powerful approach to create novel high quality drug-like molecules (Blundell et al., 2002; Murray and Rees, 2009; Murray and Blundell, 2010; Mashalidis et al., 2013). The low molecular weights of fragments (MW < 300) facilitate recognition of hotspots where binding compensates for the loss of rotational and translational entropy of the ligand by increasing entropy of “unhappy” waters displaced (Radoux et al., 2016). This allows an efficient exploration of chemical space even with libraries of around 1000 fragments, which due to their small size, interact weakly with the target protein (affinities usually between 0.1-5 mM). Fragments tend to bind to hotspots and form well defined interactions with the target protein. These initial hits can be subsequently elaborated into larger molecules with higher affinity (Murray and Blundell, 2010).

In FBDD a variety of biochemical, biophysical and structural biology methods are exploited. Popular approaches include differential scanning fluorimetry (DSF), often known as thermal shift. This is a technique that allows for the detection of compounds that increase the melting temperature (unfolding temperature, Tm) of a target protein upon binding, by promoting protein stability (Niesen et al., 2007). DSF hits can then be confirmed by ligand-based one-dimensional ^1^H NMR spectroscopy Carr-Purcell-Meiboom-Gill (CPMG), saturation transfer difference (STD) and water ligand observed gradient spectroscopy (WaterLOGSY) (Dalvit et al., 2001; Klages et al., 2007). Functional biochemical assays can also sometimes be used as a high-throughput method to screen for inhibitors. Once hits have been identified, the 3D structures of the fragment-protein complexes are defined through X-ray crystallography (usually to better than 2.5 Å resolution). Isothermal titration calorimetry (ITC) is also used to characterize fragment-binding affinities and the thermodynamics of binding. A typical fragment-based campaign for tuberculosis is described in Mendes and Blundell (2017).

For leprosy we have used the fragment hotspot maps (Radoux et al., 2016) program to indicate binding sites on the RNA polymerase holoenzyme complex in *M. leprae* (Vedithi et al., 2020). The hotspots with a contouring score of 17 were mapped to the regions of fragment binding and at the site of entry of template DNA strand into the active center cleft of the polymerase core. The accurate prediction of sites with high propensity for donor, acceptor and apolar groups (benchmarked by overlaying the hotspot maps on the rifampin-binding site) provided insights into the characteristics of the binding site and scope for novel small molecule discovery (Cambau et al., 2018). Similar programs like SiteMap (Thomas A. Halgren, 2009) and FT Map (Kozakov et al., 2015) also aid in mapping small molecule binding sites on the protein surfaces.

**FIGURE 2:**
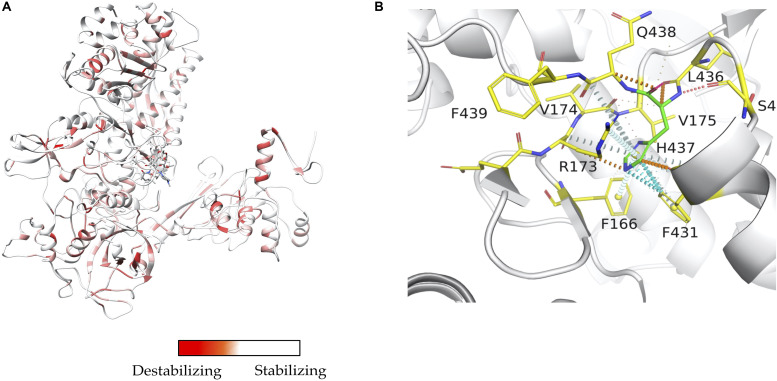
**(A)** Stability changes predicted by mCSM for systematic mutations in the ß-subunit of RNA polymerase in *M. leprae*. The maximum destabilizing effect from among all 19 possible mutations at each residue position is considered as a weighting factor for the color map that gradients from red (high destabilizing effects) to white (neutral to stabilizing effects) (Vedithi et al., 2020). **(B)** One of the known mutations in the ß-subunit of RNA polymerase, the S437H substitution which resulted in a maximum destabilizing effect [-1.701 kcal/mol (mCSM)] among all 19 possibilities this position. In the mutant, histidine (residue in green) forms hydrogen bonds with S434 and Q438, aromatic interactions with F431, and other ring-ring and π interactions with the surrounding residues which can impact the shape of the rifampin binding pocket and rifampin affinity to the ß-subunit [-0.826 log(affinity fold change) (mCSM-lig)]. Orange dotted lines represent weak hydrogen bond interactions. Ring-ring and intergroup interactions are depicted in cyan. Aromatic interactions are represented in sky-blue and carbonyl interactions in pink dotted lines. Green dotted lines represent hydrophobic interactions (Vedithi et al., 2020).

Mapping interactions between amino acid residues in protein-ligand complexes provides a better understanding of the structural implications of mutations conferring drug resistance in leprosy. We used Arpeggio (Jubb et al., 2017), an in-house developed software to map interatomic interactions. Arpeggio calculates all the intra- and inter-atomic interactions in protein-protein, protein-ligand and protein-nucleic complexes. This tool allows us to understand changes in interactions of mutant residues with the residue environment in the rifampin resistance strains of *M. leprae* (Vedithi et al., 2018; Figures 2A,B). Similar tools that aid in calculation of inter-residue interactions and energy matrices of the protein subunit interfaces are also available (Galgonek et al., 2017).

## Understanding Mechanisms of Antibiotic Resistance

Mutations can lead to antibiotic resistance not only from direct interference with drug binding but also through allosteric mechanisms that arise from mutations distant from the drug-binding site. Additionally, drug resistance can arise through mechanisms that disturb protein-protein or protein-nucleic acid interactions.

Most of the early approaches to predicting the impacts of mutations on protein structure and function were focused on the amino-acid sequence of a single protein. They included sequence-based methods such as SIFT (Ng and Henikoff, 2003) and PolyPhen (Adzhubei et al., 2010). The realization that much could be gained by knowledge of the structure of the protein led to approaches that were based on potential-energy functions or statistics of amino acid mutations with respect to the local structural environment. For example, environment-specific substitution tables, which describe the propensities of residues to mutate in a local structural environment are used in SDM (Topham et al., 1997; Worth et al., 2011; Pandurangan et al., 2017). In PoPMuSiC (Dehouck et al., 2009), and more recently BeAtMuSiC (Dehouck et al., 2013), predictions of the impact of mutations are based on multiple energy functions, with parameters trained using artificial neural networks.

Machine learning-methods have also been used to predict the impacts of mutations on protein stability from either sequence or structural features (Capriotti et al.,2005a,b; Cheng et al., 2005) and more recently to predict disease-related mutations (Capriotti and Altman, 2011). Alternatively, residue environments can be represented as graphs with atoms as nodes and interactions as edges. For example, Bongo (Cheng et al., 2008) predicts structural effects of nsSNPs using graph theoretic metrics. Graph-based structural signatures are used in mCSM for prediction of impacts of mutations on protein stability and protein-protein and protein-nucleic acid affinity (Pires et al., 2013). This approach was developed further in mCSM-lig to predict the impacts of mutations on ligand binding (Pires et al., 2016). This is based on Platinum, a database of experimentally measured effects of mutations on structurally defined protein-ligand complexes (Pires et al., 2015). This computational approach provides predictions that correlate well with experimental data (up to ρ = 0.67) in explaining Mendelian disease mutations and in predicting mutations that give rise to antibiotic resistance.

## Examples of Understanding and Combatting Resistance

The availability of whole genome sequences in the present era has greatly enhanced the understanding of emergence of drug resistance in infectious diseases like tuberculosis. The data generated by the whole genome sequencing of clinical isolates can be screened for the presence of drug-resistant mutations. A preliminary *in silico* analysis of mutations can then be used to prioritize experimental work to identify the nature of these mutations.

### Tuberculosis

We have used this combination of computational and experimental approaches in our recent studies on tuberculosis. We utilized programs developed in our lab, SDM (Pandurangan et al., 2017) and mCSM (Pires et al., 2013, 2016) to predict the effects of mutations linked to INH and rifampin resistance. These were derived from whole genome sequencing of 98 clinical isolates from Southern India (Munir et al., 2019).

**FIGURE 3:**
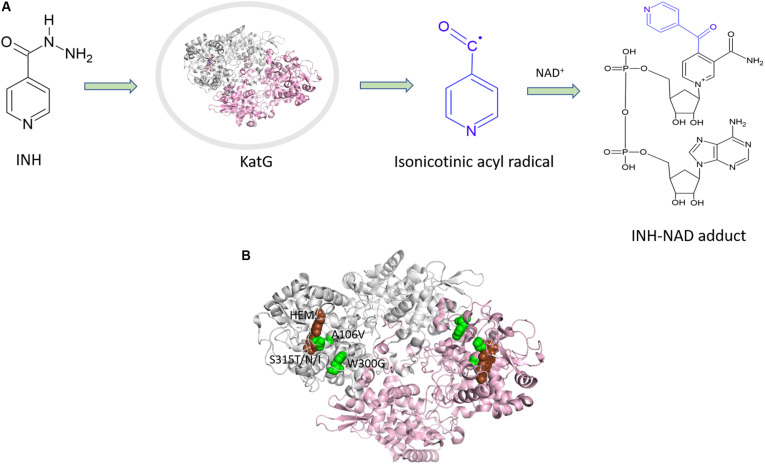
**(A)** Mechanism of isoniazid activation and INH-NAD adduct formation. **(B)** Mutations mapped (Munir et al., 2019) on the structure of KatG (PDB ID:1SJ2; Bertrand et al., 2004).

INH is a pro-drug, which is activated by the haem-dependent catalase peroxidase, KatG. Following activation, the drug creates an INH-NAD adduct (Figure 3A), which binds to InhA, an enoyl-acyl carrier protein reductase, and inhibits the synthesis of mycolic acids (Figure 3). InhA is an important enzyme in the fatty acid synthase II pathway of *M. tuberculosis*. Mutations in the KatG and InhA are responsible for the mechanism of resistance to INH. We have mapped the resistance mutations in KatG and InhA onto their protein structures and predicted the effects on protein stability, protein-protein interactions and protein-drug interactions using SDM and mCSM (Munir et al., 2019).

**FIGURE 4:**
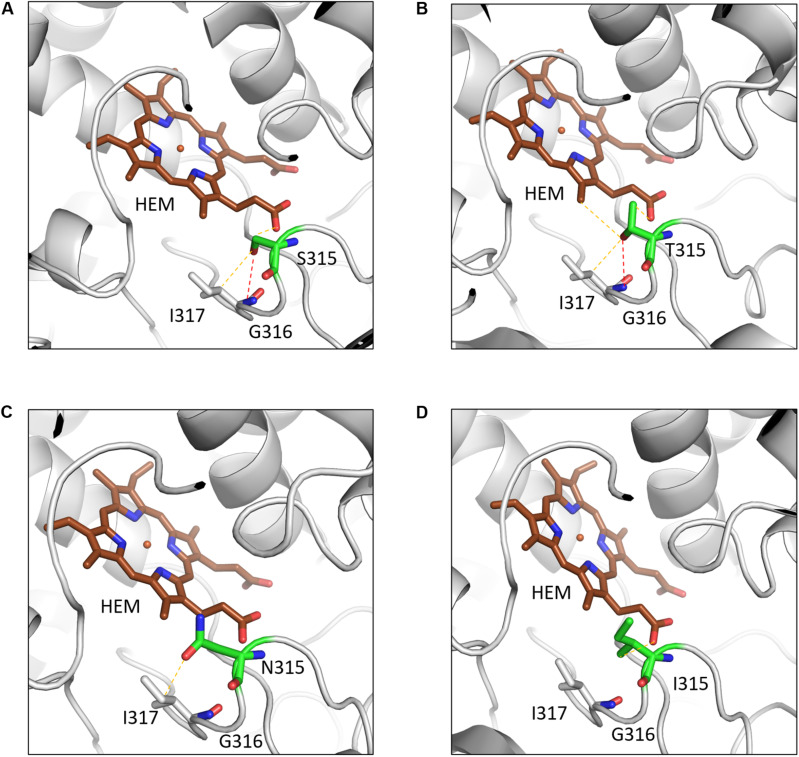
The interatomic interactions in KatG formed by the wild-type residue S315 and the mutants S315T, S315N and S315I. **(A)** The wildtype residue S315 of KatG forms a hydrogen bond (shown as red dotted lines) with the main chain of I317 and a weak hydrogen bond (orange dotted lines) with haem (HEM) and sidechain of I317. **(B)** The mutant residue T315 gains an additional weak hydrogen bond and hydrophobic interactions with haem. **(C)** The mutant residue loses the hydrogen bond with I317 and a weak hydrogen bond with haem. **(D)** The hydrophobic sidechain of isoleucine retains the weak hydrogen bond with HEM and gains hydrophobic interaction with haem (Reproduced with permission from Munir et al., 2019).

A common mutation found in KatG is S315T, which is present in more than 60% of the isoniazid-resistant isolates. This mutation has been shown previously to constrict the entrance leading to the haem active site (Zhao et al., 2006). Other mutations include S315N/I, and W300G. All of these mutations in KatG are located in the N-terminal domain of the protein, which comprises the haem-binding active site of the enzyme (Figure 3B). We found using computational approaches that these mutations affect the stability of the protein and the interatomic interactions in the local surrounding environment of the mutant residue (Figure 4). The substitution of W300 to a glycine resulted in the loss of all the hydrophobic, carbon-pi, atom-pi and weak hydrogen bond interactions made by the wild-type tryptophan with the surrounding residues. We have also recently carried out experimental work on several of these resistance mutations that surround the haem pocket in KatG. We have utilized X-ray crystallography, cryo-EM and biophysical methods to characterize the mutations and demonstrated the affects that they have on protein stability and haem binding. Among the isoniazid-resistant mutations in InhA, three mutations (S94A, I194T and I21T) were mapped onto the structure. All three mutations were located around the drug-binding pocket. The mutation S94A was predicted to decrease the affinity of drug binding to the protein, hence causing resistance to the drug. It has also been shown previously to cause a reduction in the NADH affinity and affect drug binding (Dias et al., 2007). The mutations I194T and I21T resulted in the loss of hydrophobic interactions made by the wild-type isoleucine with the surrounding residues and were predicted by mCSM-lig to decrease the drug binding affinity.

To understand the drug-resistance mechanism for rifampin, we mapped the mutations onto the RpoB (the target for rifampin), and RpoC subunits of the RNA polymerase assembly (Munir et al., 2019). Mutations in RpoB were located in the rifampin -binding pocket and S450L was the most common rifampin-resistant mutation occurring in 52% of the rifampin-resistant isolates. This mutation was predicted by mCSM-lig to decrease the affinity of the drug toward the protein, see Munir et al. (2019). The interatomic analysis clearly showed that the mutation causes the loss of a hydrogen bond formed between the drug and S450 with substitution to the bulkier side chain of leucine causing a steric clash with the drug. This likely decreases the affinity of the drug toward the protein. Other mutations in RpoB included D435Y/V, H445R/Y, S428R, V359A, S441P, L452P, and L449Q. These mutations were also found to alter the interactions either with the drug or the surrounding residues, and were predicted by mCSM-lig to decrease the affinity of the drug toward the protein. We also analyzed the impact of four mutations (L516P, N416T, V483G, and I491T) on RpoC, which are located at the interfaces with other subunits in RNA polymerase complex (Munir et al., 2019). These mutations were predicted to have a destabilizing impact on protein stability and protein-protein interactions.

Overall, in our analyses, we showed that the mutations not only affect the stability and drug binding affinity but might also act through allosteric mechanisms arising at the protein-protein interfaces.

### Leprosy

Drug resistance in leprosy continues to be a significant health problem in endemic countries. While some of the burden is overt in patients with clinical signs of relapse and/or non-responsiveness to multidrug therapy, there is a high likelihood for undetected extant strains of rifampin-resistant *M. leprae* in the leprosy communities. Computational simulations indicate that the mechanisms of rifampin resistance in leprosy and tuberculosis are similar (Vedithi et al., 2018). In the absence of an experimental method to culture *M. leprae* in the lab, drug resistance in leprosy is determined by *in vivo* propagation in mouse footpads (Levy and Ji, 2006) and by associating mutations in the drug-resistance-determining regions of the target coding genes with clinical manifestations. Missense mutations noted in rifampin, dapsone and ofloxacin-resistant strains of *M. leprae* are associated with clinical drug-resistance outcomes in leprosy (Williams et al., 2013).

Elucidating structural impacts of point mutations in the drug target proteins and their influence on function of the drug targets in mycobacteria is vital to understanding molecular mechanisms of drug resistance. We used a suite of in-house-developed tools initially to study the impacts of a few known mutations that confer rifampin resistance in leprosy (Vedithi et al., 2018). Later we used a saturation mutagenesis model where every residue in the protein is mutated to all other 19 possibilities and evaluated for protein stability, stability of the protein-protein interfaces, and affinity of the protein for nucleic acids and ligands. We then used the machine learning software, mCSM, mCSM-PPI, and mCSM-NA (Pires et al., 2013) and mCSM-lig (Pires et al., 2016), together with the statistical approach SDM (Pandurangan et al., 2017; Pandurangan and Blundell, 2020) and external tools including Maestro (Laimer et al., 2015), CUPSAT (Parthiban et al., 2006), I-Mutant (Capriotti et al., 2005a), DynaMut (Rodrigues et al., 2018) and FoldX (Schymkowitz et al., 2005). Most of these tools predict changes in Gibbs free energy of the system (△△G in kcal/mol), with the exception of mCSM-lig, which calculates the log change in affinity. For all the 40 experimentally identified rifampin-resistant mutations in *M. leprae*, mCSM-lig predicted a decrease in affinity between rifampin and RNA polymerase complex. In the saturation mutagenesis model, we have also noted similar findings in the entire conserved region of the active site in the ß sub-unit of RNA polymerase (Vedithi et al., 2020).

## Discussion

In this review, we have discussed the value of understanding the structure of drug targets in *M. tuberculosis* and *M. leprae*, first in designing new drugs and second in understanding the impacts of mutations that give rise to antimycobacterial resistance. In an earlier review article of ours (Waman et al., 2019), we discussed various computational approaches and experimental strategies for drug target identification, structure-guided drug discovery and understanding the structural implications of the mutations conferring antimicrobial resistance in mycobacterial diseases. In this review, our focus is directed toward reviewing the application of these computational tools and experimental approaches in the context of mycobacterial drug discovery and antimicrobial drug resistance with emphasis on tuberculosis and leprosy. This can then provide a greater understanding of how we may in the future redesign currently available drugs and how we may develop new ones.

In this respect the saturation mutagenesis approach, described above for the RNA polymerase complex in *M. lepra*e, enables an understanding of the mutational landscape of a protein. Of particular value is the provision of insights into which regions around the drug-binding site will less easily accept mutations and therefore less likely to experience the emergence of drug resistance mutations. This should be a useful factor to take into account during drug redesign. With respect to the RNA polymerase such regions and their relationship to fragment hotspots allow identification of two novel small-molecule binding sites. An approach like this can facilitate novel drug discovery to treat resistant strains of *M. leprae* and *M. tuberculosis* (Vedithi et al., 2020).

## Data Availability Statement

All datasets discussed in this article are available for research purposes.

## Author Contributions

TB developed the overview of the review. SV, AM, and TB prepared the manuscript and with AC revised the manuscript. All authors contributed to the article and approved the submitted version.

## Conflict of Interest

The authors declare that the research was conducted in the absence of any commercial or financial relationships that could be construed as a potential conflict of interest.
